# 靶向CD123抗原的双特异性抗体的构建及其抗急性髓系白血病的作用研究

**DOI:** 10.3760/cma.j.cn121090-20231123-00272

**Published:** 2024-03

**Authors:** 彤 周, 曼玲 陈, 楚悦 张, 晓雨 刘, 珍珍 王, 海燕 邢, 克晶 唐, 征 田, 青 饶, 敏 王, 建祥 王

**Affiliations:** 1 中国医学科学院、北京协和医学院血液病医院（中国医学科学院血液学研究所），血液与健康全国重点实验室，国家血液系统疾病临床医学研究中心，细胞生态海河实验室，天津 300020 State Key Laboratory of Experimental Hematology, National Clinical Research Center for Blood Diseases, Haihe Laboratory of Cell Ecosystem, Institute of Hematology & Blood Diseases Hospital, Chinese Academy of Medical Sciences & Peking Union Medical College, Tianjin 300020, China; 2 天津医学健康研究院，天津 301600 Tianjin Institutes of Health Science，Tianjin 301600, China

**Keywords:** 白血病，髓系，急性, CD123, 双特异性抗体, 免疫治疗, Leukemia, myeloid, acute, CD123, Dual-specific antibody, Immunotherapy

## Abstract

**目的:**

构建一种新的靶向CD123抗原的双特异性抗体（CD123 DuAb），研究CD123DuAb在急性髓系白血病（AML）治疗中的作用。

**方法:**

以自主研发的CD123单克隆抗体可变区为基础，利用分子克隆技术，构建CD123 DuAb表达质粒，转染ExpiCHO-S细胞，表达该双功能抗体。通过功能实验，验证CD123 DuAb在T细胞活化及增殖中的作用，及其促进T细胞对AML细胞的杀伤作用。

**结果:**

①构建了CD123 DuAb表达质粒，并通过Expi-CHO真核系统表达。②CD123 DuAb可以分别与T细胞上的CD3位点及CD123阳性肿瘤细胞上的CD123位点结合。③将1 nmol/L CD123 DuAb加入T细胞与MV4-11细胞共培养体系中，T细胞中CD69阳性表达率为68.0％，CD25阳性表达率为44.3％，均显著高于对照组（*P*值均<0.05）。④在CD123 DuAb浓度为1 nmol/L的条件下培养T细胞，可促进T细胞增殖，其绝对计数第1天为5×10^5^/ml，第9天扩增至3.2×10^6^/ml，且CFSE荧光强度显著降低。⑤CD123 DuAb能够显著激活T细胞，且激活强度与其浓度呈正相关，浓度为1 nmol/L时，T细胞CD107a表达率可达16.05％，较对照组显著升高（*P*<0.05）。⑥随培养体系中CD123 DuAb浓度的升高，T细胞耗竭和凋亡也随之增加，浓度为1 nmol/L时，T细胞CD8^+^PD-1^+^LAG-3^+^比例为10.90％，PI^−^Annexin Ⅴ^+^比例为18.27％，PI^+^Annexin Ⅴ^+^比例为11.43％，均较对照组显著升高（*P*<0.05）。⑦CD123 DuAb能够促进T细胞分泌细胞因子，浓度为1 nmol/L时，共培养体系上清中的IFN-γ和TNF-α的浓度可分别达到193.8 pg/ml和169.8 pg/ml，较对照组显著升高（*P*<0.05）。⑧将CD123 DuAb以1 nmol/L的浓度加入T细胞与CD123阳性肿瘤细胞共培养体系中，能显著增强T细胞对肿瘤细胞的杀伤作用，共培养3 d，CD123^+^MV4-11细胞、CD123^+^Molm13细胞和CD123^+^THP-1细胞的残留率分别为7.4％、6.7％和14.6％，较对照组显著降低（*P*<0.05）。

**结论:**

本研究构建及表达了一种靶向CD123的双特异性抗体，并通过体外实验验证其可以同时结合CD123阳性肿瘤细胞和T细胞，促进T细胞的活化和增殖，增强其抗白血病作用，为进一步临床研究提供了基础。

## Abstract (en)

**Objective:**

To construct a novel dual-specific antibody targeting human CD123（CD123 DuAb）and study its effects in acute myeloid leukemia（AML）.

**Methods:**

Based on the variable region of the CD123 monoclonal antibody independently developed at our institution, the CD123 DuAb expression plasmid was constructed by molecular cloning and transfected into ExpiCHO-S cells to prepare the antibody protein. Through a series of in vitro experiments, its activation and proliferation effect on T cells, as well as the effect of promoting T-cell killing of AML cells, were verified.

**Results:**

① A novel CD123 DuAb plasmid targeting CD123 was successfully constructed and expressed in the Expi-CHO eukaryotic system. ②The CD123 DuAb could bind both CD3 on T cells and CD123 on CD123^+^ tumor cells. ③When T cells were co-cultured with MV4-11 cells with addition of the CD123 DuAb at a concentration of 1 nmol/L, the positive expression rates of CD69 and CD25 on T cells were 68.0% and 44.3%, respectively, which were significantly higher than those of the control group（*P*<0.05）. ④Co-culture with CD123 DuAb at 1 nmol/L promoted T-cell proliferation, and the absolute T-cell count increased from 5×10^5^/ml to 3.2×10^6^/ml on day 9, and CFSE fluorescence intensity decreased significantly. ⑤ With the increase in CD123 DuAb concentration in the culture system, T-cell exhaustion and apoptosis increased. When the CD123 DuAb was added at a concentration of 1 nmol/L to the culture system, the proportion of CD8^+^ PD-1^+^ LAG-3^+^ T cells was 10.90%, and the proportion of propidium iodide（PI）^−^ Annexin Ⅴ^+^ T cells and PI^+^ Annexin Ⅴ^+^ T cells was 18.27% and 11.43%, respectively, which were significantly higher than those in the control group（*P*<0.05）. ⑥ The CD123 DuAb significantly activated T cells, and the activation intensity was positively correlated with its concentration. The expression rate of CD107a on T cells reached 16.05% with 1 nmol/L CD123 DuAb, which was significantly higher than that of the control group（*P*<0.05）. ⑦The CD123 DuAb promoted cytokine secretion by T cells at a concentration of 1 nmol/L, and the concentration of IFN-γ and TNF-α in the supernatant of the co-culture system reached 193.8 pg/ml and 169.8 pg/ml, respectively, which was significantly higher than that of the control group（*P*<0.05）. ⑧When CD123 DuAb was added at a concentration of 1 nmol/L to the co-culture system of T cells and CD123^+^ tumor cells, the killing intensity of T cells significantly increased, and the residual rates of CD123^+^ MV4-11 cells, CD123^+^ Molm13 cells, and CD123^+^ THP-1 cells were 7.4%, 6.7%, and 14.6% on day 3, respectively, which were significantly lower than those in the control group（*P*<0.05）.

**Conclusion:**

In this study, a novel CD123 DuAb was constructed and expressed. In vitro experiments verified that the DuAb binds to CD123^+^ tumor cells and T cells simultaneously, promotes T-cell activation and proliferation, and facilitates their anti-leukemia effect, which provides a basis for further clinical research.

急性髓系白血病（AML）的发生和发展与造血干细胞的成熟分化障碍、恶性克隆有关，受累的白血病细胞会出现增殖失控、分化障碍、凋亡受阻，骨髓正常造血功能受损和功能衰竭[1]。虽然常规化疗取得了一定的成效，但是AML的治疗仍然面临着难以根除白血病干细胞、复发率高、化疗毒性难以耐受等问题[2]。

近年来涌现了许多针对AML的免疫治疗方式，如单克隆抗体、抗体-药物缀合物、嵌合抗原受体T细胞（CAR-T细胞）治疗等[3]–[6]。CD123是IL-3受体的α链，与CD131形成异二聚体，构成活性IL-3受体复合物，在大约80％的AML细胞中表达，并且在白血病干细胞中富集[7]。因此，以CD123为靶点进行免疫治疗，可以有效解决常规疗法中患者难以耐受化疗毒性及复发问题。本实验室前期制备了靶向CD123的CAR-T细胞，体内外实验证实了其特异性杀伤CD123^+^AML的作用[8]–[9]。鉴于CAR-T细胞制备周期较长，越来越多的多功能抗体药物被研究和开发[10]–[12]，为临床应用提供新的思路。在本研究中，我们构建了一种新的靶向CD123的双特异性抗体（CD123 DuAb），通过一系列功能实验，验证了该CD123 DuAb促进T细胞在杀伤CD123阳性肿瘤细胞中的作用，为今后的临床研究提供理论依据。

## 材料与方法

一、主要材料及试剂

人淋巴细胞培养液KBM-581购于美国Corning公司，RPMI 1640培养基及胎牛血清购于美国Gibco公司。CFSE染料购于美国Thermo Fisher公司，流式细胞术抗体如CD69-PE、CD25-PE、CD107a-PE等购自美国Biolegend公司。ELISA试剂盒购于南京金斯瑞生物有限公司；蛋白脱盐及浓缩柱购于美国Millipore公司；ExpiCHO-S细胞、ExpiCHO 瞬时转染试剂、His tag蛋白纯化柱等购于美国Thermo Fisher公司。莫能霉素购于德国Sigma公司，RossetteSep T细胞富集试剂购于美国Stem Cell公司，细胞因子联合检测试剂盒购于杭州赛基生物。MV4-11、Molm13及K562细胞系购自ATCC并于本实验室保存，健康成人外周血标本取自天津市血液中心。

二、细胞培养

使用含10％胎牛血清的RPMI 1640培养基培养人AML细胞系MV4-11细胞、Molm13细胞；使用ExpiCHO表达培养基培养ExpiCHO-S细胞; 用Ficoll淋巴细胞分离液及RossetteSep T细胞富集试剂富集人T细胞，并使用含有50 U IL-2和5％胎牛血清的KBM-581培养基培养。

三、双特异性抗体的表达与纯化

在pcDNA3.4载体的基础上构建CD123 DuAb表达质粒，按照使用手册对ExpiCHO细胞进行转染，并在转染后第1、5天分别加入适当量辅料，培养后离心收集上清。应用Western blot法验证上清中产生的抗体，并使用AKTA purifie蛋白纯化系统及His蛋白纯化柱对收取的上清进行纯化。使用Millipore超滤柱对上清进行浓缩，并用PBS置换，将浓缩脱盐后的蛋白保存于−80 °C冰箱。使用考马斯亮蓝染色验证蛋白纯度，使用His tag ELISA试剂盒对其进行定量。

四、双特异性抗体功能鉴定

1. 双特异性抗体结合特异性鉴定：取三管MV4-11细胞分别设置为实验组、阴性对照组和阳性对照组，先在实验组和阴性对照组分别加入1 nmol/L CD123 DuAb和PBS，再将anti-CD123单克隆抗体分别加入实验组、阳性对照组和阴性对照组中，常温避光，孵育15 min；使用PBS清洗并重悬，采用流式细胞术检测荧光强度。采用相同方法检测该CD123 DuAb与T细胞表面CD3抗原结合的特异性。

2. T细胞增殖检测：

（1）绝对计数：将人T细胞以5×10^5^/ml的密度接种于6孔板中，分别在实验组和对照组加入1 nmol/L CD123 DuAb及PBS，在37 °C、5％ CO_2_环境中培养，并每隔3 d进行绝对计数，记录T细胞增殖情况。

（2）T细胞分裂鉴定：使用CFSE染料对人T细胞进行染色，并将染色后的T细胞以2.5×10^5^/ml的密度接种于96孔板中，并在对照组和实验组中分别加入PBS及不同浓度的CD123 DuAb（0.01、0.1、1 nmol/L），在共培养72 h后采用流式细胞术检测T细胞的荧光强度，评估T细胞的增殖情况。

3. T细胞活化检测：将人T细胞与MV4-11细胞以效应细胞∶靶细胞（E∶T）＝5∶1共培养，并在对照组和实验组中分别加入PBS及不同浓度的CD123 DuAb（0.01、0.1、1 nmol/L），在共培养24 h和48 h后分别使用anti-CD69、anti-CD25单克隆抗体进行标记，流式细胞术检测T细胞中CD69及CD25表达的比例。

4. T细胞耗竭检测：将T细胞以5×10^5^/ml的密度接种于96孔板中，并在对照组和实验组中分别加入PBS及不同浓度的CD123 DuAb（0.01、0.1、1 nmol/L），在24 h后采用流式细胞术检测T细胞中CD8^+^PD-1^+^LAG-3^+^细胞比例，评估T细胞的耗竭情况。

5. T细胞凋亡检测：将T细胞以5×10^5^/ml的密度接种于96孔板中，并在对照组和实验组中分别加入PBS及不同浓度的CD123 DuAb（0.01、0.1、1 nmol/L），在24 h后采用流式细胞术检测T细胞的PI^−^Annexin V^+^比例及PI^+^Annexin V^+^比例，评估T细胞的早期及晚期凋亡情况。

6. T细胞脱颗粒检测：将人T细胞与MV4-11细胞以E∶T＝5∶1共培养，并在对照组和实验组中分别加入PBS及不同浓度的CD123 DuAb（0.01、0.1、1 nmol/L），同时加入anti-CD107a单克隆抗体进行标记，共培养1 h后加入莫能霉素，继续培养4 h后，使用流式细胞术检测T细胞表面CD107a的表达，评估T细胞的脱颗粒情况。

7. T细胞分泌细胞因子的检测：将人T细胞与MV4-11细胞以E∶T＝5∶1共培养，并在对照组和实验组中分别加入PBS及不同浓度的CD123 DuAb（0.01、0.1、1 nmol/L），在共培养72 h后3 000 r/min离心3 min（有效离心半径为86 mm）收取上清，将收取的上清与细胞因子检测试剂盒中的微球室温孵育2 h，PBS洗涤，流式细胞术检测，并使用FCAP（3.0.1）软件进行分析，评估上清中细胞因子的分泌情况。

8. T细胞杀伤功能实验：使用CFSE染料对人T细胞进行染色，使用APC膜染色染料对MV4-11细胞进行染色，将染色后的人T细胞与MV4-11细胞以E∶T＝5∶1共培养，并在对照组和实验组中分别加入PBS及不同浓度的CD123 DuAb（0.01、0.1、1 nmol/L），共培养72 h后采用流式细胞术检测残留靶细胞的比例。以同样的方式检测T细胞与Molm13细胞系共培养中CD123 DuAb介导的T细胞杀伤功能。

五、统计学处理

每组实验均进行3次生物学重复，采用GraphPad Prism 8 软件对数据进行统计学分析，所有的数据均以均数±标准差表示，对于两组以上的比较采用one-way ANOVA检验。*P*<0.05为差异具有统计学意义。

## 结果

一、CD123 DuAb的构建和表达

1. CD123 DuAb表达质粒的构建：以本实验室已有的CD123单克隆抗体可变区及CD3单克隆抗体可变区为基础，串联两者构建成为CD123scFv-CD3scFv序列，连接至pcDNA3.4载体，成功构建pcDNA3.4-CD123 DuAb真核表达质粒，载体结构如图1A。

2. CD123 DuAb的表达：在Expi-CHO细胞中转染pcDNA3.4-CD123 DuAb真核表达质粒，通过Western blot法检测，在相对分子质量55×10^3^处可见蛋白条带，且未见其他条带（图1B），提示使用Expi-CHO细胞成功表达了CD123 DuAb蛋白。使用Alphafold2对蛋白结构进行预测，结果如图1C。培养适当时间后收获上清并进行纯化、浓缩和脱盐，以获得较纯的双特异性抗体蛋白。

**图1:**
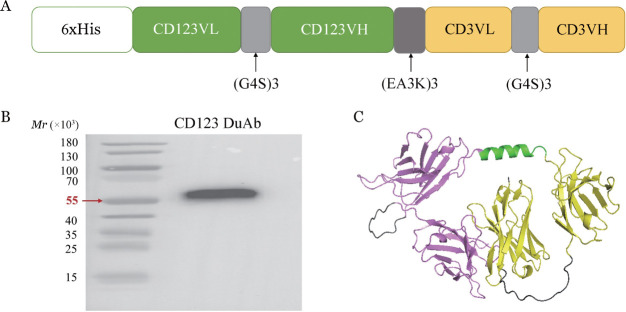
靶向CD123的双特异性抗体（CD123 DuAb）的构建及表达 A CD123 DuAb载体构建示意图；B Western blot法验证CD123 DuAb的表达；C Alphafold2预测CD123 DuAb结构

二、CD123 DuAb的亲和特异性

通过CD123 DuAb与商品化抗体PE-anti-CD3（HIT3a）以及APC-anti-CD123（6H6）的竞争结合实验，发现CD123 DuAb能够竞争结合至人T细胞上的CD3抗原（图2A）及MV4-11细胞的CD123抗原（图2B），表明我们构建的CD123 DuAb保留了与CD3抗原及CD123靶抗原的特异性结合能力。

**图2:**
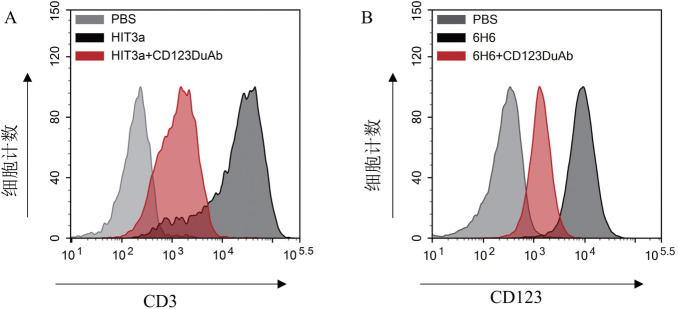
靶向CD123的双特异性抗体（CD123 DuAb）结合特异性分析 A CD123 DuAb与CD3单克隆抗体（HIT3a）竞争结合T细胞上的CD3抗原；B CD123 DuAb与CD123单克隆抗体（6H6）竞争结合T细胞上的CD123抗原

三、CD123DuAb对人T细胞的表型的影响

1. CD123 DuAb促进人T细胞的增殖：相比于PBS对照组，加入1 nmol/L CD123DuAb共孵育的实验组中，T细胞绝对计数明显增加，培养至第9天时，T细胞绝对计数由初始的5×10^5^/ml扩增至3.2×10^6^/ml，提示CD123 DuAb能够有效刺激T细胞的增殖（图3A）。

**图3:**
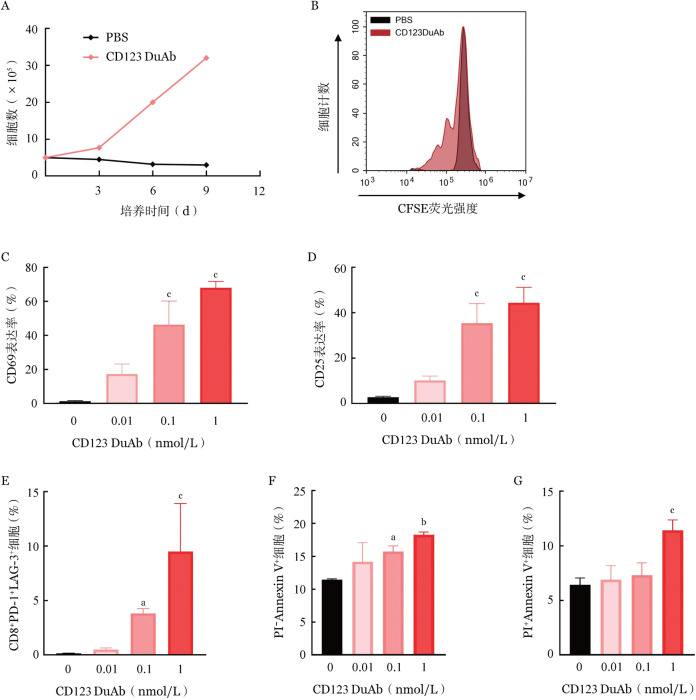
靶向CD123的双特异性抗体（CD123 DuAb）对T细胞表型的改变（实验重复3次） A CD123 DuAb促进T细胞增殖；B CD123 DuAb促进T细胞分裂；C CD123 DuAb促进T细胞CD69表达；D CD123 DuAb促进T细胞CD25表达；E CD123 DuAb对T细胞耗竭的影响；F CD123 DuAb影响T细胞早期凋亡；G CD123 DuAb影响T细胞晚期凋亡 注 与PBS对照组（0 nmol/L）比较，^a^*P*<0.05、^b^*P*<0.01、^c^*P*<0.001

使用CFSE标记T细胞后继续培养3 d，相比于PBS对照组，加入1 nmol/L CD123 DuAb共孵育的实验组中，随着T细胞增殖峰的出现，CFSE标记的T细胞荧光强度逐渐下降（图3B），提示T细胞在CD123 DuAb刺激下增殖、分裂，导致CFSE荧光随其增殖减弱。

2. CD123 DuAb对人T细胞的活化作用：将不同浓度的CD123 DuAb与人T细胞以及MV4-11靶细胞共孵育24 h，T细胞CD69表达率随CD123 DuAb浓度增高而增加，CD123 DuAb浓度为1 nmol/L时，CD69阳性率达68.0％，显著高于对照组（图3C）。共孵育48 h，T细胞CD25表达率随CD123 DuAb浓度增高而增加，CD123 DuAb浓度为1 nmol/L时，CD25阳性率为44.3％，显著高于对照组（图3D）。

3. CD123 DuAb影响人T细胞的耗竭：将不同浓度的CD123 DuAb与人T细胞共孵育24 h，T细胞CD8^+^PD-1^+^LAG-3^+^比例随CD123 DuAb浓度增高而增加，提示T细胞耗竭比例增加（图3E）。

4. CD123 DuAb影响人T细胞的凋亡：将不同浓度的CD123 DuAb与人T细胞共孵育24 h，T细胞PI^−^Annexin Ⅴ^+^比例随CD123 DuAb浓度增高而增加，提示T细胞早期凋亡比例增加（图3F）；T细胞PI^+^Annexin Ⅴ^+^比例随CD123 DuAb浓度增高而增加，提示T细胞晚期凋亡比例增加（图3G）。

四、CD123 DuAb能够有效促进T细胞分泌细胞因子

收集T细胞与MV4-11靶细胞共培养72 h后的上清，与细胞因子检测磁珠共孵育，流式细胞术检测显示，上清中IFN-γ和TNF-α的浓度随CD123 DuAb浓度增加而逐步升高，在CD123 DuAb浓度为1 nmol/L的实验组中，IFN-γ和TNF-α的浓度可分别达到193.8 pg/ml（图4A）和169.8 pg/ml（图4B），显著高于PBS对照组，表明CD123 DuAb能够有效促进T细胞细胞因子的分泌。

**图4:**
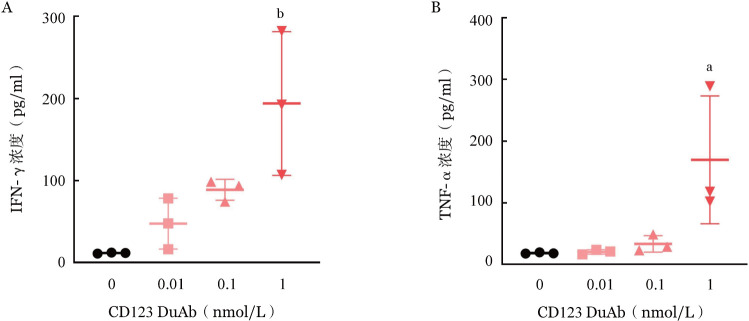
靶向CD123的双特异性抗体（CD123 DuAb）促进T细胞分泌细胞因子 A CD123 DuAb促进T细胞分泌IFN-γ；B CD123 DuAb促进T细胞分泌TNF-α 注 与PBS对照组（0 nmol/L）比较，^a^*P*<0.05，^b^*P*<0.01

五、CD123 DuAb能够介导人T细胞对CD123阳性肿瘤细胞的特异性杀伤作用

以CD123^+^MV4-11细胞为靶细胞，将CFSE染色后的人T细胞与APC膜染色后的靶细胞MV4-11以E∶T＝5∶1混合，加入不同浓度的融合蛋白，共培养72 h，流式细胞术检测人靶细胞的残留率，结果显示靶细胞残留率随融合蛋白浓度增高而降低，浓度为1 nmol/L时，靶细胞残留率仅为7.4％。用同样的方法，分别以CD123^+^Molm13细胞和CD123^+^THP-1细胞为靶细胞，同样显示了CD123 DuAb对Molm13靶细胞的有效杀伤（图5A～F），表明CD123 DuAb能够介导人T细胞对表达CD123的肿瘤细胞的特异性杀伤。

在脱颗粒实验中，我们在T细胞与CD123^+^MV4-11细胞共培养体系中加入不同浓度的CD123 DuAb，共培养4 h后采用流式细胞术检测T细胞中CD107a表达情况，结果显示T细胞的CD107a表达比例与CD123 DuAb浓度呈正相关，当CD123 DuAb浓度为1 nmol/L时，CD107a可达16.05％，表明该双特异性抗体能够介导T细胞的激活（图5G）。

**图5:**
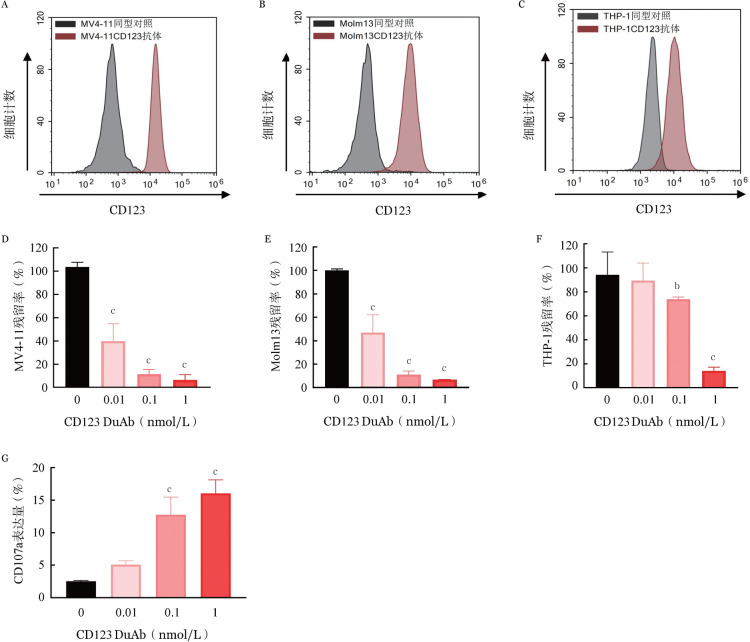
靶向CD123的双特异性抗体（CD123 DuAb）促进T细胞对CD123阳性肿瘤细胞的杀伤作用（实验重复3次） A MV4-11细胞CD123表达；B Molm13细胞CD123表达；C THP-1细胞CD123表达；D CD123 DuAb促进T细胞对MV4-11细胞的裂解作用；E CD123 DuAb促进T细胞对Molm13细胞的裂解作用；F CD123 DuAb促进T细胞对THP-1细胞的裂解作用；G CD123 DuAb促进T细胞脱颗粒作用 注 与PBS对照组（0 nmol/L）比较，^a^*P*<0.05，^b^*P*<0.01，^c^*P*<0.001

## 讨论

AML是一种以骨髓和外周血的髓系原始细胞克隆性增殖并导致造血功能障碍为特征的疾病，目前AML的治疗仍然以常规化疗及异基因造血干细胞移植为主。然而许多患者不能耐受强化疗，且部分患者经标准方案治疗后疗效欠佳，发展为复发难治性（R/R）AML[13]。因此，为了改善R/R AML患者的预后，寻找安全有效的全新治疗方法迫在眉睫。

目前针对AML的免疫治疗主要包括CAR-T细胞治疗和抗体治疗等。然而，CAR-T细胞治疗存在制备时间长、成本高昂等问题，而双特异性抗体则可以作为一种工业化批量制备的产品，即取即用，为患者提供更加及时的免疫治疗。本实验室在前期的研究中成功构建了一种靶向CD19的双特异性抗体及增强型双特异性抗体，并通过体内及体外实验成功验证其功能[14]–[15]。

CD123是IL-3受体的α亚单位，越来越多的临床证据表明，CD123表达于原始细胞，尤其是在白血病干细胞（LSC）中富集，在LSC上的表达水平明显高于正常造血干细胞，并与AML患者的预后密切相关。此外，研究发现CD123在初诊和复发时的白血病细胞和LSC中均高表达，其表达模式不受白血病细胞克隆演变的影响[16]。因此，CD123被认为是治疗AML的潜在靶点，靶向CD123的免疫疗法也引起了广泛的研究兴趣，迄今为止，已有多个以CD123为靶点的免疫治疗方式被开发，本实验室前期制备的CD123 CAR-T细胞也具有较好的疗效[9]。本研究中，我们构建了一种新的靶向CD123的双特异性抗体，并通过体外实验证实其能够发挥对肿瘤细胞的杀伤作用，其在体内的杀伤作用有待进一步研究。在体外实验中，我们发现随着抗体浓度的提高，T细胞的耗竭和凋亡比例也随之增加，该发现也提示了在后续的研究中，应当对该双特异性抗体的应用浓度进行更进一步的探索验证，以平衡双特异性抗体对T细胞活化与耗竭的影响。除AML细胞外，CD123也在其他多种恶性血液肿瘤细胞，如慢性淋巴细胞白血病和急性淋巴细胞白血病中高表达[17]–[18]，因此，该双特异性抗体在肿瘤治疗中的应用潜力值得进一步探索。此外，一些研究发现，CAR-T细胞或抗体药物免疫治疗呈现显著疗效的同时，也伴随着细胞因子释放综合征等多种不良反应的产生，从而引起高热、肌痛等症状，甚至危及生命[19]–[22]，在后续的研究中，我们也将对此持续关注，通过摸索应用条件与更改结构等方式避免此类问题的发生。

综上所述，我们构建了一种新的靶向CD123和CD3的双特异性抗体，该双特异性抗体能够分别与T细胞上的CD3和肿瘤细胞上的CD123靶点结合，促进T细胞的活化和增殖，提高T细胞分泌细胞因子水平，促进T细胞对肿瘤细胞的杀伤作用，为进一步临床研究提供了基础。

## References

[1] Tyner JW, Tognon CE, Bottomly D (2018). Functional genomic landscape of acute myeloid leukaemia[J]. Nature.

[2] Abdel-Aziz AK (2023). Advances in acute myeloid leukemia differentiation therapy: A critical review[J]. Biochem Pharmacol.

[3] Tabata R, Chi S, Yuda J (2021). Emerging Immunotherapy for Acute Myeloid Leukemia[J]. Int J Mol Sci.

[4] Baroni ML, Sanchez Martinez D, Gutierrez Aguera F (2020). 41BB-based and CD28-based CD123-redirected T-cells ablate human normal hematopoiesis in vivo[J]. J Immunother Cancer.

[5] Xu S, Zhang M, Fang X (2021). A novel CD123-targeted therapeutic peptide loaded by micellar delivery system combats refractory acute myeloid leukemia[J]. J Hematol Oncol.

[6] Mardiros A, Dos Santos C, McDonald T (2013). T cells expressing CD123-specific chimeric antigen receptors exhibit specific cytolytic effector functions and antitumor effects against human acute myeloid leukemia[J]. Blood.

[7] Shi M, Su RJ, Parmar KP (2019). CD123: A Novel Biomarker for Diagnosis and Treatment of Leukemia[J]. Cardiovasc Hematol Disord Drug Targets.

[8] 王 珍珍, 卢 杨, 徐 颖茜 (2020). 一种新的CD123嵌合抗原受体T细胞的构建及其抗白血病作用探究[J]. 中华血液学杂志.

[9] Wang Z, Lu Y, Liu Y (2023). Novel CD123×CD33 bicistronic chimeric antigen receptor (CAR)-T therapy has potential to reduce escape from single-target CAR-T with no more hematotoxicity[J]. Cancer Commun (Lond).

[10] Gauthier L, Morel A, Anceriz N (2019). Multifunctional Natural Killer Cell Engagers Targeting NKp46 Trigger Protective Tumor Immunity[J]. Cell.

[11] Wu L, Seung E, Xu L (2020). Trispecific antibodies enhance the therapeutic efficacy of tumor-directed T cells through T cell receptor co-stimulation[J]. Nat Cancer.

[12] Sivori S, Pende D, Quatrini L (2021). NK cells and ILCs in tumor immunotherapy[J]. Mol Aspects Med.

[13] Döhner H, Weisdorf DJ, Bloomfield CD (2015). Acute Myeloid Leukemia[J]. N Engl J Med.

[14] Chen M, Liu X, Peng N (2023). Construction of CD19 targeted dual- and enhanced dual-antibodies and their efficiency in the treatment of B cell malignancy[J]. Exp Hematol Oncol.

[15] 陈 曼玲, 彭 楠, 刘 晓雨 (2021). 一种新的靶向CD19抗原的三特异性T细胞衔接器的制备及其抗白血病作用研究[J]. 中华血液学杂志.

[16] Ehninger A, Kramer M, Röllig C (2014). Distribution and levels of cell surface expression of CD33 and CD123 in acute myeloid leukemia[J]. Blood Cancer J.

[17] Nievergall E, Ramshaw HS, Yong AS (2014). Monoclonal antibody targeting of IL-3 receptor α with CSL362 effectively depletes CML progenitor and stem cells[J]. Blood.

[18] Muñoz L, Nomdedéu JF, López O (2001). Interleukin-3 receptor alpha chain (CD123) is widely expressed in hematologic malignancies[J]. Haematologica.

[19] Akhtar OS, Sheeba BA, Azad F (2024). Safety and efficacy of anti-BCMA CAR-T cell therapy in older adults with multiple myeloma: A systematic review and meta-analysis[J]. J Geriatr Oncol.

[20] Hernani R, Benzaquén A, Solano C (2022). Toxicities following CAR-T therapy for hematological malignancies[J]. Cancer Treat Rev.

[21] Sun T, Li D, Huang L (2023). Inflammatory abrasion of hematopoietic stem cells: a candidate clue for the post-CAR-T hematotoxicity?[J]. Front Immunol.

[22] Suntharalingam G, Perry MR, Ward S (2006). Cytokine storm in a phase 1 trial of the anti-CD28 monoclonal antibody TGN1412[J]. N Engl J Med.

